# Extraction of Sensitive Bands for Monitoring the Winter Wheat (*Triticum aestivum*) Growth Status and Yields Based on the Spectral Reflectance

**DOI:** 10.1371/journal.pone.0167679

**Published:** 2017-01-06

**Authors:** Chao Wang, Meichen Feng, Wude Yang, Guangwei Ding, Lujie Xiao, Guangxin Li, Tingting Liu

**Affiliations:** 1 Dryland Farming Engineer Institute, Shanxi Agricultural University, Taigu, China; 2 Department of Chemistry, Northern State University, Aberdeen SD, United States of America; 3 Institute of Crop Science, Shanxi Academy of Agricultural Sciences, Taiyuan, China; Murdoch University, AUSTRALIA

## Abstract

To extract the sensitive bands for estimating the winter wheat growth status and yields, field experiments were conducted. The crop variables including aboveground biomass (AGB), soil and plant analyzer development (SPAD) value, yield, and canopy spectra were determined. Statistical methods of correlation analysis, partial least squares (PLS), and stepwise multiple linear regression (SMLR) were used to extract sensitive bands and estimate the crop variables with calibration set. The predictive model based on the selected bands was tested with validation set. The results showed that the crop variables were significantly correlated with spectral reflectance. The major spectral regions were selected with the B-coefficient and variable importance on projection (VIP) parameter derived from the PLS analysis. The calibrated SMLR model based on the selected wavelengths demonstrated an excellent performance as the R^2^, TC, and RMSE were 0.634, 0.055, and 843.392 for yield; 0.671, 0.017, and 1.798 for SPAD; and 0.760, 0.081, and 1.164 for AGB. These models also performed accurately and robustly by using the field validation data set. It indicated that these wavelengths retained in models were important. The determined wavelengths for yield, SPAD, and AGB were 350, 410, 730, 1015, 1185 and 1245 nm; 355, 400, 515, 705, 935, 1090, and 1365 nm; and 470, 570, 895, 1170, 1285, and 1355 nm, respectively. This study illustrated that it was feasible to predict the crop variables by using the multivariate method. The step-by-step procedure to select the significant bands and optimize the prediction model of crop variables may serve as a valuable approach. The findings of this study may provide a theoretical and practical reference for rapidly and accurately monitoring the crop growth status and predicting the yield of winter wheat.

## Introduction

The traditional method for obtaining the physiological and biochemical parameters of crops is mainly based on taking physical samples from the fields, and then measuring them by using chemical methods in the lab. However, it is time consuming, labor intensive, and destructive [1]. The non-destructive or non-intrusive approach by using hyperspectral technology can provide an efficient tool to overcome these problems. This technology has been also proved to be effective in rapidly estimating crop growth status, grain yield, and quality [2]. Crop canopy sensors were commonly used in precision agriculture to estimate agronomic parameters including chlorophyll, AGB, and plant nitrogen abundance (or deficiency) [3–6]. The simple statistical analysis always suffers the problem of over-fitting because the number of spectral band far exceeds the sample size [7]. Previous studies indicated that vegetation indexes combined visible and near-infrared bands can minimize spectral noise, and correlate to the crop growth variables and crop physiological parameters [8, 9]. The extraction of the sensitive bands that mainly contain hyperspectral information of crop variables is the foundation and the premise for constructing the vegetation index [10]. Recognizing and/or extracting some sensitive spectral bands from numerous wavelengths is very crucial step for overcoming the over-fitting and collinear problems and improving the model accuracy [11]. However, determining the central or sensitive wavelengths for establishing predictive model is often a challenge [12, 13]. The selection of new wavebands in hyperspectral imaging has been carried out in a number of cases that mainly focused on how to increase sensitivity of the vegetation index to chlorophyll, nitrogen content, and other physical parameters [14]. Optimizing multiple narrow bands by using stepwise linear regression analysis has been commonly used to identify the important bands related to plant nitrogen status [15]. However, only depending on the SMLR method could not significantly improve the generality of prediction model [9]. Thus, many researchers have adopted mathematical technique and statistical procedure to effectively mine the ill-posed hyperspectral data and overcome the over-fitting problems [16]. Vasques et al [17] successfully extracted the sensitive bands of soil organic matter to realize its accurate prediction using the stepwise multiple linear regression (SMLR) method. Li et al. [18] tested the performance of spectral indicators and partial least squares (PLS) method to compare their accuracy in predicting canopy nitrogen content of winter wheat. They further reported that PLS was a potentially useful approach in deriving canopy nitrogen content of winter wheat under the conditions of different growth stages and different cultivars when numerous kinds of canopy reflectance data were included in the calibration models. Previous investigation also proved that the PLS was an effective method in mining hyperspectral data during the model development process [19], selecting significant bands [20], and overcoming the problems of collinearity and over-fitting [21]. The PLS could be used to provide a useful exploratory and predictive tool in analyzing hyperspectral data and extracting hyperspectral information [12, 22].

The leaf chlorophyll content, nitrogen content, and AGB are important crop parameters because they can show the vegetative growth status [23, 24]. It is crucial to accurately and quickly evaluate the growth status and grain yield with the hyperspectral technology [25]. Numerous studies found a close correlation between SPAD value and nitrogen content, leaf chlorophyll content. They further recommended that the SPAD readings could be made as a nitrogen status indicator [26, 27]. Wang et al. [28] reported that the SPAD value could be estimated with a high coefficient of determination (R^2^ = 0.7444, RMSE = 7.359) by using the method of continuous wavelet transformation.

In order to deal with the complicated phenomenon of high dimensionality and redundancy in processing of hyperspectral data, it is essential to extract the sensitive bands for estimating the winter wheat growth status and grain yield. More specifically, the objectives of the research were to: (i) extract the significant band information representing growth status indicator and yield of winter wheat, and overcome the high dimensionality and redundancy of hyperspectral data with the method of multivariate analysis, (ii) effectively evaluate the growth status and predict yield using extracted sensitive bands, and (iii) explore a feasible approach to extract the sensitive bands of growth status indicator and yield of winter wheat.

## Materials and Methods

### Experiments design

Experiment (Exp.) 1: The experiment was carried out at the experimental station (N 37°25', E 112°33') of Shanxi Agriculture University (P. R. China) from September of 2011 to July of 2012. The climate in local area belongs to arid area with an average annual rainfall of 440 mm and mean annual temperature of 11°C. The soil of the field was classified as a Calcareous Cinnamon soil developed from loess parent material (Alfisols in U.S. taxonomy) with 22.01 g kg^-1^ organic matter, 53.8 mg kg^-1^ total N, 18.43 mg kg^-1^ available phosphate, and 236.9 mg kg^-1^ available potassium. The winter wheat cultivar of Jing 9549 was sown in September of 2011 with a planting density of 6 million plants per hectare. The experiment was a randomized complete block design with three replications. For each treatment, the nitrogen rates: 0 kg ha^-1^ (N0), 100 kg ha^-1^ (N1), 200 kg ha^-1^ (N2), 300 kg ha^-1^ (N3), and 400 kg ha^-1^ (N4) were applied for each plot at pre-sowing basal and at jointing stage with the ratio of 6:4 [29]. For all treatments, calcium phosphate and potassium chloride were applied as basal dose at 120 kg ha^-1^ (P_2_O_5_) and 150 kg ha^-1^ (K_2_O), respectively. The experiment plot was 20 (4 by 5) square meters and the routine field management was conducted as usual. All plot measurement including canopy spectra, SPAD value, and AGB was mainly made at reviving, jointing, heading, and filling stages. All data obtained from three replications for the same treatment were averaged. Total 20 sample data were obtained, and these data were used as the calibration set.

Experiment (Exp).2: The experiment was conducted in the same area with *Exp*. 1 from September in 2012 to July in 2013. It was a split plot randomized complete block design with three replications. Three varieties of winter wheat (Jing 9549, Chang 4738, and Jinnong 190) were assigned as the main plot and five nitrogen application rates (0 kg ha^-1^, 75 kg ha^-1^, 150 kg ha^-1^, 225 kg ha^-1^, and 300 kg ha^-1^) were applied as the sub-plot treatments, with an area of 20 (4 by 5) square meters for each plot. The same amounts of P_2_O_5_ and K_2_O as of experiment 1 were applied. The same field management of *Exp*. 1 was carried out in *Exp*. 2. All samples were mainly taken at jointing, heading, and filling stages. 15 sample data were achieved for each growth stage and total 45 samples were also designed as the calibration set.

Experiment (Exp).3: The experiment was conducted in Wenxi County (N 34°35'-35°49', E 110°13'-112°4'), China, where the majority of the winter wheat located in flatland and minority on the hill, with an average annual rainfall of 740 mm and mean annual temperature of 14°C. At heading stage of winter wheat, 20 sample sites including the irrigation wheat fields and non-irrigation wheat fields owned by local farmers were randomly selected. The farmland size was less than 0.5 hectare throughout the county. Therefore, the growth status of winter wheat for 20 sample sites had a wide range due to the various winter wheat varieties, diverse fertilizer applications, and different routine field managements. The experiment was used to further confirm the accuracy of selected sensitive bands through validating the application and robustness of SMLR models for crop variable. This experiment was initiated as the validation set.

### Measurement of SPAD value, AGB, and yield

For each measurement of all crop growth indicators and canopy spectra, more than 2 m^2^ of the winter wheat that had a consistent growth status were selected in each plot. The samples were taken at each growth stage. The 1 m^2^ of the winter wheat was used for obtaining the spectral reflectance, SPAD value, and AGB; and another 1 m^2^ was used to measure the grain yield at harvesting period.

SPAD value: The SPAD value was determined by using a SPAD instrument (Soil and Plant Analyzer Development, Japan, 502) on the top second leaf. The SPAD values were evenly measured for 9 times from the leaf sheath to leaf apex in each leaf. Five leaves were randomly selected and measured. All the SPAD readings were averaged as the final SPAD value.

AGB (10^4^ kg ha^-1^): 1 square meter of winter wheat plant samples was taken after the measurement of canopy spectra and SPAD value. The sample was weighed in lab and the mass of the sample was determined.

Yield (kg ha^-1^): At harvesting stage, the grain yield was determined at each target point (1 m^2^) in each of the growth stage.

### Measurement of canopy reflectance

The canopy reflectance of winter wheat was obtained with an ASD spectroradiometer (Analytical Spectral Devices, Inc. (ASD), USA) under cloudless conditions and as close to solar noon as possible. The ASD spectrometer is operated in the 350–2500 nm spectral region, with a sampling interval of 1.4 nm and spectral resolution of 3 nm between 350 and 1050 nm; and a sampling interval of 2 nm and spectral resolution of 10 nm between 1050 and 2500 nm. The viewing angle was set at 25°. In the target area of winter wheat, three target points were selected and ten spectra were obtained for each point. These measurements were then averaged as the final spectrum for the target area. The canopy of winter wheat height was 1 m. Prior to the measurement of canopy reflectance in each plot, a standard whiteboard (Labsphere, North Sutton, NH, USA) was used to calibrate the spectral reflectance.

### Pre-process of hyperspectral reflectance and crop variables

The raw spectral reflectance obtained from the spectrometer always contains background information and noise. Therefore, it is essential to process the raw spectral reflectance by eliminating the abnormal spectrum, averaging the same canopy spectrum, and splicing correction. Then, the spectral reflectance was smoothed with 8 points in a Savitzky-Golay way to eliminate the effect of noise and background information [30]. In current study, we mainly paid attention to the spectral region of 350–1400 nm that contained most of crop growth status information. Every 5 wavebands was averaged into one spectral band variable to reduce the hyperspectral dimension for all hyperspectral bands [31]. Eventually, 1051 wavebands were reduced to 211.

### Multivariate analysis

#### Correlation analysis

The correlation coefficient analysis that can illustrate the relationship between two variables is a common method to extract the sensitive bands from 2151 wavelengths that are always collinear and redundant. Previous studies [9, 19, 27, 28] reported that the sensitive bands always appeared in the region where there was a large correlation coefficient or the correlation coefficient rapidly shifting. These spectral regions are always deemed to contain much more variable information.

#### PLS analysis

The PLS method is a technique that generalizes and/or combines the features of principal component analysis and multiple regressions [32]. It is particularly useful when PLS is used to predict a set of dependent variables from a large set of independent variables since it can overcome the co-linearity and realize the dimension reduction for hyperspectral bands. It will be not losing much hyperspectral information [33]. Thus, the result of PLS analysis is also used for selecting the sensitive band regions [34, 35].

Selecting the optimal factor number is one of the most important processes in PLS analysis. The B-coefficient and variable importance on projection (VIP) derived from PLS analysis are also important parameters. B-coefficient expresses the correlation between independent and dependent variable, and represents the importance and influence of independent variables on dependent variables. The independent variables with larger B-coefficients can always be viewed as a large contribution to the predictive model. However, Lee [36] thought that the sensitive bands regions could not be selected based on B-coefficient parameters alone. VIP parameter is another variable to show the distribution and effect of independent variable to the PLS model. Wold [37] reported that the independent variables could be eliminated if the B-coefficient parameter is lower and VIP value is less than 0.8, simultaneously.

#### SMLR analysis

The SMLR method combines a forward selection and a backward elimination. Initially, the independent variable is imported to the regression equation based on the influence, distribution, and the significance of dependent variable as affected by independent variable. Then, the best variable that has a higher coefficient at the significant probability level (α = 0.05) in each step is added. Furthermore, all variables that enter the regression are checked to see if any variables will be removed using the significant criterion (α = 0.01). The next independent variable will be imported and the process will stop if no more variables can be imported or eliminated. The independent variable that enters the model is closely related to the dependent variable. Vasques et. al. [17] reported that it was a potential method to analyze and select useful hyperspectral wavelengths.

### Procedures of sensitive band extraction

To clearly show how to extract the sensitive bands of crop variable in the paper, the four steps were initiated.

1. Correlation analysis: The correlation analysis was conducted between the SPAD, AGB, yield variable, and canopy spectral variable by using the calibration set. The results would provide reference for the extracted wavelengths derived from multivariate analysis.
2. PLS analysis: The PLS models of crop variables were established under the optimal factor number by using the calibration set, and then the sensitive band regions were determined with the B-coefficient and VIP parameters derived from PLS model.
3. SMLR analysis: The sensitive band regions were input to select the sensitive bands and the predictive models were constructed based on the selected wavelengths by using the calibration set.
4. Validation: The *Exp*. 3 was applied to confirm the accuracy of selected sensitive bands through validating the application and robustness of SMLR models.

### Validation parameters

The validation was based on the parameters of theil coefficient (TC), root mean squared error (RMSE) [37]. Their equations were defined by:

$$TC=\frac{\sqrt{\frac{1}{t}{\sum_{t=1}^{t}\left(\overset{\wedge }{y_{t}}-y_{t}\right)}^{2}}}{\sqrt{\frac{1}{t}{\sum_{t=1}^{t}\left(\overset{\wedge }{y_{t}}\right)}^{2}}+\sqrt{\frac{1}{t}{\sum_{t=1}^{t}\left(y_{t}\right)}^{2}}}\;(t=1,2,3\ldots \;t)$$

$$RMSE=\sqrt{\frac{1}{t}{\sum_{t=1}^{t}\left(\overset{\wedge }{y_{t}}-y_{t}\right)}^{2}}\;(t=1,2,3\ldots \;t)$$

Where, t is the sample number, and ${\overset{\wedge }{y}}_{t}$, *y*_*t*_ is the predictive value and measured value, respectively. The TC value ranges from 0 to 1, indicating that the smaller number for TC, the better predictive effectiveness for predicted value and measured value.

## Results

### Statistical analysis of crop variable

In this study, to accurately extract the important wavelengths that are sensitive to the grain yield, SPAD, and AGB, the experiment 1 and 2 (including four cultivars under different years, various nitrogen application rates and managements, together with different sample periods) were merged into the calibration set (Table 1). The data in Table 1 shows that the range and SD of three crop variables were wide. It indicated that the calibration set created a wide range variation of growth status (SPAD and ABG) and grain yield. This might simulate the performance of winter wheat growth and hyperspectral reflectance in practice as realistic as possible. Moreover, the validation set derived from the field experiment 3 also held a similar range and a larger SD value if comparing the results with calibration set. The experiment 3 would confirm the accuracy of selected sensitive bands through validating the application and robustness of SMLR models for crop variables.

**Table 1:** Statistical analysis of yield, SPAD, and aboveground biomass (AGB) of winter wheat for three experiments.

	Crop variables	N	Range	Min	Max	Average	SD
Calibration set (Experiment 1 and 2)	Yield (kg ha^-1^)	65	5917.964	4549.171	10467.136	7596.864	1045.343
	SPAD	65	14.116	48.184	62.300	53.171	3.161
	AGB (10^4^ kg ha^-1^)	65	8.183	3.590	11.773	6.870	2.392
Validation set (Experiment 3)	Yield (kg ha^-1^)	20	5568.554	4687.524	10256.077	7500.574	1305.994
	SPAD	20	13.678	48.622	62.300	52.872	3.958
	AGB (10^4^ kg ha^-1^)	20	8.177	3.590	11.767	7.373	2.744

Note: N, sample number; SD, standard deviation; Min, minimum; Max, maximum; The unit for yield and AGB is kg ha^-1^ and 10^4^ kg ha^-1^, respectively. SPAD is dimensionless.

### Analysis of canopy reflectance under different conditions

**Fig 1:**
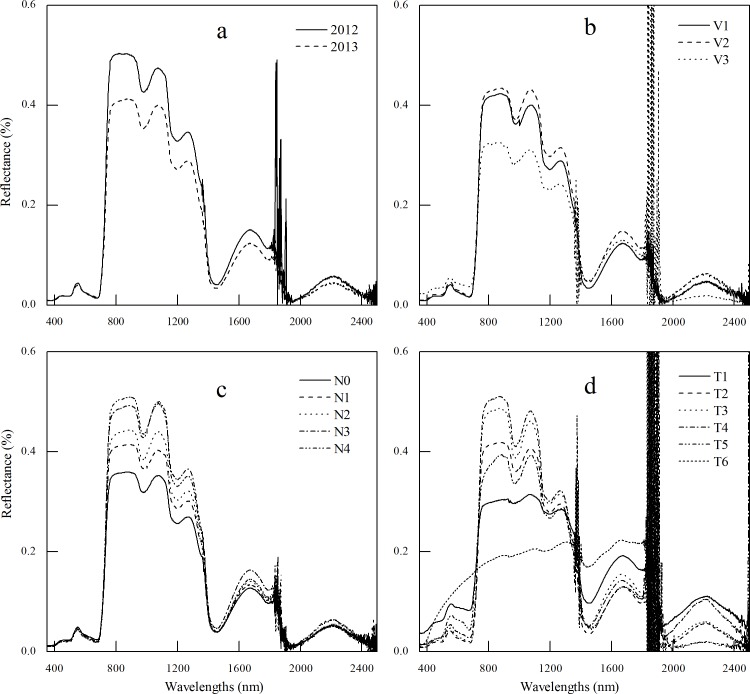
Canopy reflectance of winter wheat under different experimental treatments. **(a)** described the canopy reflectance for the variety of Jing 9549 at jointing stage in 2012 and 2013; **(b)** showed the canopy reflectance for different varieties in 2013 (V1, V2, and V3 were the variety of Chang 4738, Jing 9549, and Jinnong 190, respectively); **(c)** was the canopy reflectance for Jing 9549 under different nitrogen levels (introduced in *Exp*. 1); **(d)** was the canopy reflectance for Jing 9549 at different growth and development periods (T1, T2, T3, T4, T5, and T6 are green stage, jointing stage, booting stage, flowering stage, filling stage, and maturity stage, respectively).

The data (Fig 1A) showed that the spectral reflectance in visible band as affected mainly by chlorophyll [38] was similar to the same variety of winter wheat. However, there was an obvious difference in near-infrared band affected by the inner structure of leaf and the growth status of winter wheat. There was a large difference for the spectral reflectance between three varieties of winter wheat, especially for the variety of Jinnong 190 (Fig 1B). The spectral reflectance did not change in a large degree in visible band with the application of nitrogen. However, it increased in near-infrared band where the increased rate gradually dwindled and the spectral saturated phenomenon occurred [15, 39] (Fig 1C). The data (Fig 1D) demonstrate that the spectral reflectance in visible band decreased at first and then increased with the growth and development period of winter wheat until at mature stage when the wave crest and wave trough disappeared. Moreover, the reflectance increased at first and then decreased to the lowest at mature stage. Then, the double-peak disappeared that was the typical characteristics of plant. Overall, the hyperspectral reflectance sensibly responded to the wide variation of growth status. This sensitivity might provide the possibility to extract the hyperspectral information of crop variables.

**Fig 2:**
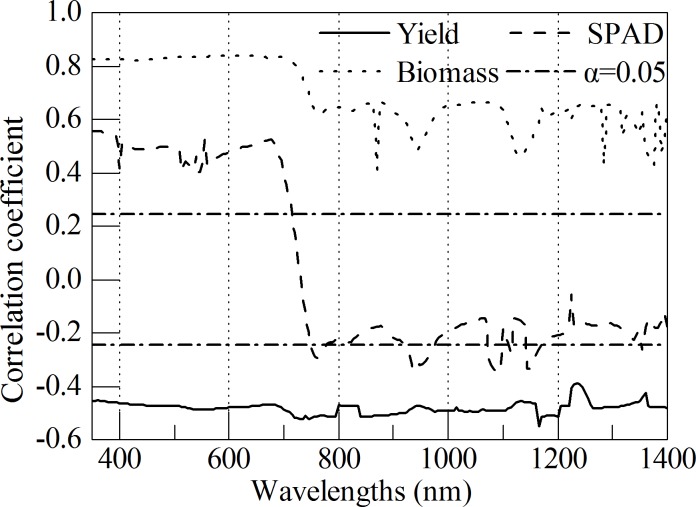
Correlation analysis between growth status indicators, yield, and spectral reflectance of winter wheat.

The correlation coefficients were analyzed between crop variables and the spectral wavelengths by using the calibration set as shown in Fig 2. The Fig 2 illustrates that there was a significantly positive and negative band region from 350–1400 nm for AGB and yield of winter wheat, respectively. The SPAD was positively correlated with these wavelengths from 350 to 740 nm. A negatively correlation with the wavelength region of 740–1400 nm was observed. For the SPAD and AGB variables, the correlation coefficient noticeably shifted in red edge region (680–760 nm). The correlation coefficient for SPAD and AGB in 350–680 nm was high and some similar peaks located at these wavelengths: 680, 760, 870, 940, 11230, 1230, and 1355 nm. The close correlation between SPAD and AGB resulted in the fact that the feature of correlation coefficient was similar. Although the negative correlation coefficient was lower than 0.6 for grain yield, it also passed the significant test at the 0.05 level. Its correlation coefficient in visible region was stable. However, obvious variance in the red edge and near infrared regions, especially at these wavelengths: 730, 940, 1160, 1240, and 1360 nm was documented. Considering the fact that the sensitive bands always hold a close correlation with crop variables, the correlation analysis would provide some valuable reference to compare or validate the spectral bands extracted with the method of multivariate analysis.

### PLS analysis

**Fig 3:**
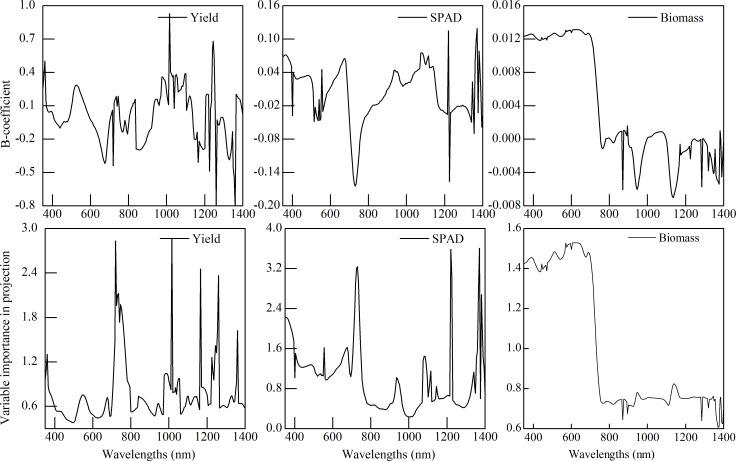
Selection of sensitive spectral region for the yield, SPAD, and aboveground biomass (AGB) of winter wheat based on the B-coefficient and VIP parameters.

A desirable model should have a high R^2^, low TC, and RMSE, as well as less number of variables. In current study, PLS models for three crop variables (grain yield, SPAD, and AGB) were initiated under different number of factors; while only the most accurate models with the parameters of TC, RMSE, and R^2^ were selected in Table 2. The data in Table 2 indicate that the optimal factor numbers were 8, 5, and 2 for the PLS models of yield, SPAD, and AGB, respectively. The calibrated and validated models for three crop variables had a moderated performance (Table 2). Simultaneously, the B-coefficient and VIP parameter derived from PLS analysis are shown in Fig 3. The Fig 3 presents that the peaks for B-coefficient and VIP were similar. The wavelength that holds a high VIP always shows a large B-coefficient at the same time. Based on the selective principle that the VIP exceeded 1 (0.8 for biomass as the VIP in near infrared was thoroughly lower than 1), the absolute value of B-coefficient parameter was higher at the same time. The sensitive band regions of yield, SPAD, and AGB variables are selected and presented in Table 3. These bands demonstrated either a shift or a significant peak.

**Table 2:** Extraction of optimal factor numbers for hyperspectral PLS models of yield, SPAD, and aboveground biomass (AGB).

Crop variables	Calibrated model			Validated model			Optimal factor number
	R^2^	TC	RMSE	R^2^	TC	RMSE	
Yield	0.6939	0.05007	771.5222	0.7376	0.043422	663.9267	8
SPAD	0.7159	0.015699	1.671999	0.736	0.046444	4.724085	5
AGB	0.7076	0.088983	1.28344	0.8851	0.090443	1.338688	2

Note: TC, theil coefficient; RMSE, root mean squared error; R^2^, determination coefficient.

**Table 3:** Extraction of sensitive band region based on the B-coefficient and VIP parameter of PLS method.

Crop variables	Range 1 (nm)	Range 2 (nm)	Range 3 (nm)	Range 4 (nm)	Range 5 (nm)
Yield	350–395	700–795	925–1055	1165–1260	-
SPAD	350–555	580–750	935	1075–1115	1335–1395
AGB	350–740	895	1155–1225	1285	1320–1365

### Extraction of sensitive bands for SPAD, AGB, and grain yield based on SMLR method

**Fig 4:**
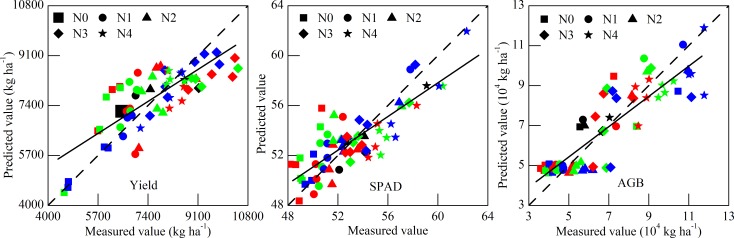
The SMLR models of yield, SPAD, and AGB using the calibration set. The dashed line and solid line was 1:1 line as a reference and fitted line between the measured value and predicted value, respectively. The filling color of black, red, green, and blue represents the reviving stage, jointing stage, heading stage, and filling stage, respectively.

**Fig 5:**
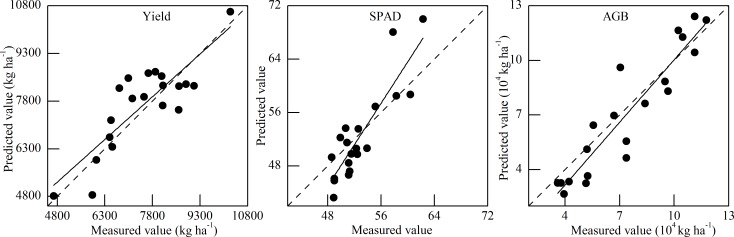
The SMLR models of yield, SPAD, and AGB using the validation set. The dotted line and solid line was 1:1 line as a reference and fitted line between the measured value and predicted value, respectively.

The sensitive band regions as the independent variable are categorized to SMLR analysis to extract the important hyperspectral bands. The extracted sensitive bands of SPAD, AGB, and grain yield are listed in Table 4. The performance of SLMR models for three variables is shown in Fig 4. The distance from the sample position to the fitting lines in coordinate axis was calculated to show the effect of growth stage under same nitrogen treatment on calibrated model (Table 5). Moreover, the SMLR models of crop variable were validated using the validation parameters (R^2^ and RMSE) to further confirm the accuracy of selected sensitive bands with the validation set conducted under complex eco-climate situation differing with other two experiments. The Table 6 shows the performance of calibrated models and validated models for three crop variables. The fitted effect between measured values and predicted values is illustrated in Fig 5. The Table 6 illustrates that the calibrated SMLR models based on the selected wavelengths had a good performance as the R^2^, TC, and RMSE were 0.634, 0.055, and 843.392 for yield; 0.671, 0.017, and 1.798 for SPAD; and 0.760, 0.081, and 1.164 for AGB. These models also performed an accurate and robust (a moderate) prediction by using the field validation set (Fig 5). The parameters of R^2^, TC, RMSE were 0.714, 0.049 and 752.016 for yield; 0.787, 0.036, and 3.795 for SPAD; and 0.863, 0.086, and 1.327 for AGB.

**Table 4:** Extraction of sensitive band for yield, SPAD, and aboveground biomass (AGB) based on the method of SMLR analysis.

Crop variables	Selected sensitive bands (nm)
Yield	350, 410, 730, 1015, 1185, 1245
SPAD	355, 400, 515, 705, 935, 1090, 1365
AGB	470, 570, 895, 1170, 1285, 1355

**Table 5:** The sample distance character of different growth stages under the same nitrogen treatment from the sample position to the fitting lines in coordinate axis.

	GS1	GS2	GS3	GS4
yield	312.0444	607.9427	467.4189	362.0641
SPAD	0.7147	1.126244	0.944861	0.866495
Biomass	0.594319	0.573267	0.569123	0.762692

Note: GS1, GS, GS3, and GS4 were the reviving, jointing, heading, and filling stage of winter wheat.

**Table 6:** Validation parameters of SMLR models for yield, SPAD, and aboveground biomass (AGB) based on the extracted sensitive bands.

Crop variables	Calibrated model			Validated model			Number of wavelengths
	R^2^	TC	RMSE	R^2^	TC	RMSE	
Yield	0.634	0.055	843.392	0.714	0.049	752.016	6
SPAD	0.671	0.017	1.798	0.787	0.036	3.795	7
AGB	0.760	0.081	1.164	0.863	0.086	1.327	6

Note: R^2^, determination coefficient; RMSE, root mean squared error.

## Discussion

In this study, a large number of samples with various representations were identified and investigated to select the sensitive band related to crop variable including grain yield, SPAD, and AGB. This is an essential step for developing a real-time spectral prediction of crop growth status and grain yield. Furthermore, the correlation analysis, PLS analysis, and SMLR analysis were applied to select and extract the sensitive band regions associated with crop growth status and yield. The optimal factor number was determined with the validation parameters to establish the PLSR models. The sensitive band regions were selected with the B-coefficient and VIP parameters derived from PLS analysis. Then, the selected band regions were identified as an input to the SMLR analysis to extract the sensitive bands. The SMLR models based on the sensitive bands were validated under field experiment. Generally, if the selected independent variables retained in predictive model contain the major information of dependent variables, the approach of constructing model was in the right direction and the model of dependent variable may be accurate. However, it is still imperative that the robustness and application of the predictive model of crop variable are needed to be further validated under complex conditions, *e*.*g*., numerous varieties of winter wheat, inconsistent grow status of winter wheat, different fertilization application, and heterogeneous eco-climate region [34]. That is why the *Exp*. 3 in Wenxi County was implemented to confirm the accuracy of selected sensitive bands through validating the application and robustness of SMLR models for crop variable.

In current study, the wavelengths of 350, 410, 730, 1015, 1185, and 1245; 355, 400, 515, 705, 935, 1090, and 1365; 470, 570, 895, 1170, 1285, and 1355 nm for yield, SPAD, and AGB of winter wheat, respectively, were extracted as the most informative identification. It was noted that the spectral bands from 350 to 700 nm were important for photosynthetic capacity, which always affect the plant growth status and grain yield [40]. That was why the sensitive bands of 350, 410, and 730; 355, 400, 515, and 705; and 470 and 570, were associated with yield, SPAD, and AGB, respectively. The wavelengths of 400, 410, and 470 nm belonged to the green region. There were several reports proving the importance of the region in evaluating the plant growth due to the significant correlation between the green region and the crop variables (Fig 2) [10, 41]. The spectral region of 680–760 nm was the red edge that was proved to be effective and accurate in estimating chlorophyll content [42], total nitrogen [43], and yield [20]. Yacobi et al. [44] presented that the 713 nm wavelength was optimal to estimate chlorophyll content. Kira et al. [45] reported that the spectral band around 720 nm always remained high sensitive with chlorophyll absorption and avoided the saturation phenomenon at moderate to high chlorophyll content. Similar wavelength of 700 nm was reported to predict the SPAD in these studies [36, 46]. It seems that the 705 nm wavelength was important for estimating SPAD in the current study. The spectral region (760–1400 nm) was governed by canopy structure, leaf cell structure, and water absorption. The character of leaf and canopy was significantly related to AGB and the spectral region contained some important spectral information for AGB. Everard et al. [47] pointed out that the spectral range 880–1680 nm was more accurate than spectral region from the 450 to 950 nm indicating that these bands were informative for AGB of winter wheat. The sensitive bands of AGB were mainly documented in the spectral region in our study. Weber et al. [40] identified that spectral reflectance of unknown physiological relevance could be used to predict yield. The most informative bands of 1030, 1110, and 1260 nm were determined to monitor yield of winter wheat.

To further validate the accuracy of sensitive bands for yield, SPAD, and AGB, we compared these bands with the result of correlation analysis and PLS. If compared to wavelengths selected by multivariate analysis (Table 4) with correlative analysis (Fig 2), the conclusion could be made as the following. That is: most of the selected wavelengths was located in these regions where there were high correlation coefficients (such as, 730 and 1185 nm for yield; 935, 1090 nm for SPAD; 570 nm for AGB), coefficient peaks (such as, 730, 1015, 1185, and 1245 for yield; 400, 515, 705, 935, 1090, and 1365 nm for SPAD; 895, 1170, 1285, and 1355 nm for AGB), and/or a rapid shift of correlation coefficient (730 nm for yield and 705 nm for SPAD). For multivariate method, except the wavelengths 410 nm for yield; 935 for SPAD; and 1285 and 1355 nm for AGB, the left wavelengths in Table 4 also locates in the region where the VIP and B-coefficient were high. It is indicated that the correlation analysis and PLS analysis are also an alternative approach to determine the important wavelength. Moreover, the SMLR models based on these sensitive bands performed moderately in predicting the growth status and grain yield in winter wheat field. Therefore, it is noted that the selected spectral bands were sensitive and important with crop variables in our study.

The PLS method is widely used in spectral quantitative analysis [45, 40]. In this research, the method was applied to determine the important spectral regions and reduce the hyperspectral dimension. Moreover, the PLS models of grain yield, SPAD, and AGB were established and achieved a good performance under the optimal number of latent factors. The SMLR model based on the important wavelengths also performed moderately as more significant wavelengths were retained into the model. However, compared with the important wavelength regions selected with the PLS analysis, the number of significant wavelengths pertained in the SMLR model was further reduced. It indicated that both methods of PLS (Table 2) and SMLR (Table 6) could reduce the predictor variables. Considering the fact that SMLR models had a moderate prediction, a sensitive wavelength has more potential application in practice. The multivariate method is feasible in reducing the multi-collinearity problem, selecting the significant wavelengths, and predicting the interest variables.

The calibrated models were established in our study. The significant wavelengths were selected based on important growth stages of winter wheat for experiment 1 and 2, respectively. Thus, different sampling times would definitely affect the accuracy of calibrated model. Our result demonstrated that these samples collected at the filling stage performed a better fitting in the calibrated models of yield and SPAD; and the samples at the heading stage and jointing stage were followed (Fig 4). It indicated that the filling stage might have an effect on the calibrated models of yield and SPAD. For AGB, the best performance of samples was obtained from the heading stage, and the jointing stage and filling stage were subsequently followed. The heading stage might provide more information pertaining to AGB prediction. The sample distance character of different growth stages under the same nitrogen treatment from the sample position to the fitting lines in coordinate axis could indirectly prove the above result (Table 5).

The spectral reflectance is very sensitive to the objective characters and external factors. It was noted that in term of quantitative analysis of hyperspectrum, many factors, such as varieties of winter wheat, planting density, plant morphology, plant health status, soil background, spectral testing method, and testing conditions will ultimately affect the accuracy and application of monitor models of crop variables. In order to broad adaptability of sensitive bands and improve the robustness and applicability of spectral inversion models for crop variables, it is necessary to increase crop varieties and sample data, expand research area under different ecological climate and complex environmental factors, and introduce mathematical and multivariate analysis [13]. The field experiments in this study investigated three wheat cultivars, six N fertilization rates in two consecutive growing seasons, and different ecological environment (including in Wenxi County in 2013). Compared with previous studies [48, 49], the predictive accuracy of yield model for winter wheat could be classified as moderately reliable (*R*^*2*^ = 0.65) and acceptable for cultivation of wheat production. This might be explained by the fact that the spectral bands were reduced from 2151 to 211 and some important hyperspectral bands could be out of phase. In addition, the less number of sample data and the different region field reduced the predictive accuracy of models. Therefore, the selected sensitive band needs to be further validated. The monitor model of cop variables also requires further optimization under the various varieties of winter wheat, increased sample data, expanded region scope, and increased mathematically multivariate analysis.

## Conclusion

Previously, various studies have been conducted to select the sensitive bands, extract the spectral characteristics, and construct vegetation index using single statistical method, which may not overcome the over-fitting and co-linearity phenomenon of hyperspectral. Our current study tried to overcome the difficulties in the process and explore the reasonable approaches to solve the problems. These significant wavelengths 350, 410, 730, 1015, 1185, and 1245 nm for yield; 355, 400, 515, 705, 935, 1090, and 1365 nm for SPAD; and 470, 570, 895, 1170, 1285, and 1355 nm for AGB were determined by using the multivariate method. Moreover, SMLR models based on the selected wavelengths could moderately predict the grain yield and evaluate the growth status as the R^2^, TC, RMSE were 0.634, 0.005, and 843.392 for yield, 0.671, 0.017, and 1.798 for SPAD, and 0.760, 0.08, and 1.164 for AGB. It indicated that step-by-step procedure developed with the multivariate methods was proved to be effective in determine significant wavelengths and evaluate the growth status and yield of winter wheat. The findings of this investigation may provide theoretical and practical reference in the wheat production using hyperspectral remote sensing.
